# Dual Inhibition of GSK3 and JAK by BIO Suppresses Osteoblast Differentiation and Mineralization of Human Mesenchymal Cells

**DOI:** 10.3390/cimb48030316

**Published:** 2026-03-16

**Authors:** Nihal Almuraikhi, Latifa Alkhamees, Sumaiya Tareen, Manikandan Muthurangan

**Affiliations:** Stem Cell Unit, Department of Anatomy, College of Medicine, King Saud University, Riyadh 11461, Saudi Arabia; 442204106@student.ksu.edu.sa (L.A.); stareen@ksu.edu.sa (S.T.); mrangan@ksu.edu.sa (M.M.)

**Keywords:** GSK3 signaling, JAK/STAT signaling, human bone marrow MSCs, osteoblast differentiation

## Abstract

Glycogen synthase kinase-3 (GSK3) inhibition is a commonly used approach to promote osteogenic differentiation through activation of Wnt signaling. However, 6-bromoindirubin-3′-oxime (BIO), which is commonly used for GSK3 inhibition, also targets JAK/STAT, raising the possibility of dual pathway interference during osteoblast differentiation, as both GSK3 and JAK/STAT pathways are critical regulators of osteoblastogenesis. In this study, we investigated the effect of BIO on the osteoblast differentiation of hMSCs-TERT4. While BIO had no significant effect on cell viability or apoptosis, it markedly inhibited osteoblast differentiation, as evidenced by reduced ALP activity, decreased matrix mineralization, and downregulation of osteoblast-associated markers. Microarray analysis followed by qRT-PCR validation revealed downregulation of Wnt and TGF-β pathway genes. These findings show that BIO suppresses osteoblast commitment and osteogenic differentiation, accompanied by altered Wnt- and TGF-β-related gene expression. This study provides mechanistic insight into the off-target consequences of widely used small molecules and highlights the importance of dissecting pathway-specific roles in stem cell differentiation. Understanding the interplay between GSK3 and JAK signaling is essential for optimizing pharmacological strategies in skeletal regenerative medicine. This study highlights the importance of pathway selectivity when using small molecules in stem cell-based therapies for bone regeneration.

## 1. Introduction

**Figure 1:**
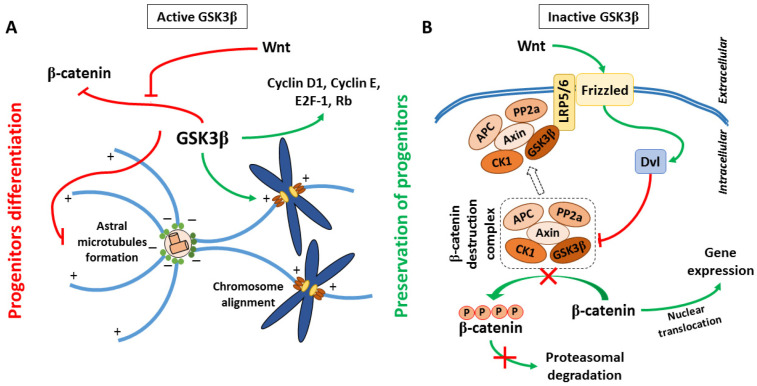
GSK3 signaling pathway. (**A**) Active GSK3β promotes β-catenin degradation and cellular differentiation. (**B**) Wnt signaling inhibits the GSK3β destruction complex, allowing β-catenin stabilization and nuclear gene activation. Reproduced from Hajka et al., 2021 [Cells 2021, 10, 2092] under Creative Commons Attribution (CC BY) license [15].

Bone remodeling is maintained through a balance between osteoblast-mediated bone formation and osteoclast-mediated resorption [1,2]. Osteoblasts originate from mesenchymal stem cells (MSCs), making their lineage commitment and differentiation vital to skeletal regeneration [3]. The process is regulated by multiple pathways, including Wnt/β-catenin, TGF-β, BMP, Hedgehog, Notch, and JAK/STAT, which together orchestrate gene transcription and matrix mineralization [4]. Among these, canonical Wnt signaling is particularly critical for osteoblast lineage commitment, acting through β-catenin–dependent induction of transcription factors such as RUNX2 and Osterix that regulate matrix deposition and mineralization [5,6]. Glycogen synthase kinase-3 (GSK3) functions as a major negative regulator of this pathway, and its inhibition stabilizes β-catenin, thereby promoting early osteogenic gene expression [7,8]. Glycogen synthase kinase-3 (GSK3) is a constitutively active serine/threonine kinase existing as two highly homologous isoforms, GSK3α and GSK3β, whose activity is regulated by site-specific phosphorylation [9]. Inhibition of GSK3 downstream of Wnt/β-catenin or insulin/PI3K–Akt signaling occurs via phosphorylation at Ser21 (GSK3α) or Ser9 (GSK3β), resulting in stabilization of β-catenin and activation of genes involved in proliferation, differentiation, and migration (Figure 1) [10,11]. Active GSK3 negatively regulates Wnt signaling by phosphorylating β-catenin to promote its degradation and modulates TGFβ signaling by phosphorylating Smad3, thereby controlling its stability and transcriptional output [8,12]. Through integration of Wnt/β-catenin, TGFβ/Smad, and PI3K/Akt/mTOR pathways, GSK3 functions as a central regulator of cellular homeostasis and osteogenic differentiation, and can be pharmacologically inhibited by ATP-competitive compounds such as BIO [13,14].

6-bromoindirubin-3′-oxime (BIO) is widely used as a potent ATP-competitive inhibitor of GSK3 and is routinely employed in stem cell research to activate Wnt signaling [16]. Because selective GSK3 inhibitors have been shown to enhance osteoblast differentiation and mineralization [5,17], BIO is often assumed to exert similar pro-osteogenic effects. However, emerging biochemical evidence demonstrates that BIO is not exclusively selective for GSK3, exhibiting affinity for several kinases involved in cytokine signaling and cell-cycle regulation [18,19]. These broader interactions raise the possibility that BIO may engage signaling mechanisms beyond canonical Wnt activation.

**Figure 2:**
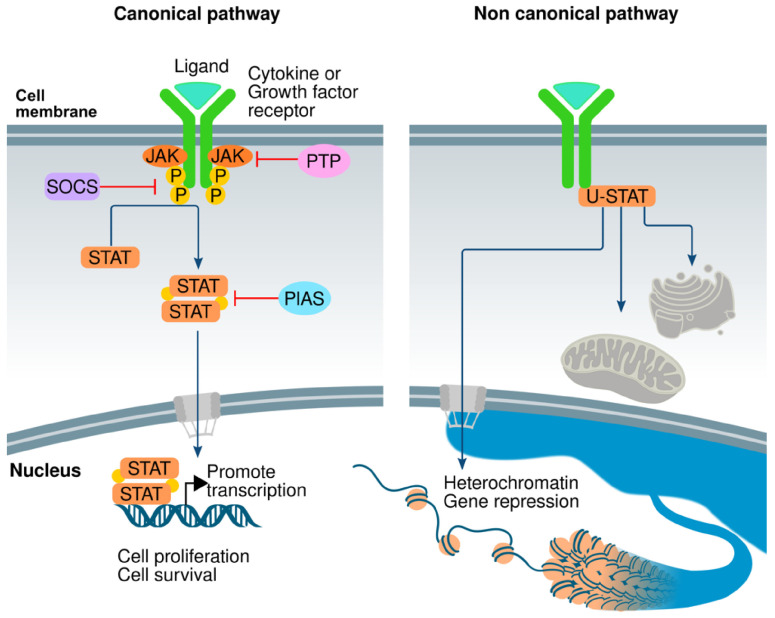
JAK/STAT signaling pathway. Reproduced from Valle-Mendiola et al., 2023 [Genes 2023, 14, 1141.] under Creative Commons Attribution (CC BY) license [24].

In parallel, increasing attention has focused on the role of Janus kinase (JAK) mediated pathways in MSC differentiation. JAKs are a family of intracellular non-receptor tyrosine kinases that play a critical role in the transduction of signals initiated by a variety of cytokines, interferons, and growth factors [20,21,22]. JAKs phosphorylate STAT proteins, causing them to undergo nuclear translocation, in turn allowing them to control gene expression in response to extracellular stimuli (Figure 2) [23]. JAK activation occurs upon ligand binding to cytokine receptors, which results in receptor dimerization and phosphorylation; this process recruits and phosphorylates STAT proteins, which translocate to the nucleus and regulate gene expression (Figure 2) [20,23].

Despite the use of BIO as a GSK3 inhibitor, relatively few studies have examined its overall impact on osteogenic differentiation under conditions where multiple pathways may be simultaneously affected. The degree to which BIO promotes or suppresses osteoblast formation, and whether its actions reflect GSK3 inhibition alone or broader kinase modulation, remains insufficiently defined.

Therefore, the present study investigates the effects of BIO on osteogenic differentiation of human MSCs and evaluates how BIO-mediated kinase modulation influences transcriptional cascades associated with osteoblast lineage progression. By integrating biochemical, functional, gene expression analyses, and bioinformatics, this work aims to clarify BIO’s net impact on osteogenesis and to reassess its suitability as a tool compound for targeting GSK3 in bone-related applications.

## 2. Materials and Methods

### 2.1. Culturing of Cells and Proliferation Medium

Human mesenchymal stem cells (hMSCs) are extracted and isolated from bone marrow [25]. A telomerase human mesenchymal stem cell line (hMSC-TERT4) is a line of cells with the overexpression of the reverse transcriptase gene (hTERT) that is commonly used as a model for hMSCs [25]. This type of cell line, hMSC-TERT4, usually provides the typical features of hMSCs, such as expressing the markers for early-stage hMSCs, multipotency, and self-renewal [26].

In this study, the hMSC-TERT4 cell line is utilized as a model for human mesenchymal stem cells (hMSCs), which exhibit the typical features of primary hMSCs.

A vial of hMSC-TERT4 passage 44 was retrieved from a −80 °C freezer and thawed using a hot bath at 37 °C. The proliferation medium used for seeding the cells consisted of Dulbecco modified eagle medium (DMEM), which was complemented by 4500 g/L D-glucose, 4 mM L-glutamine, 110 mg/L 10% sodium pyruvate, 45 mL fetal bovine serum FBS + 5 mL penicillin-streptomycin, and 1% nonessential amino acids. Reagents used in making this media were purchased from Thermo Fisher Scientific Inc. (Waltham, MA, USA) (www.thermofisher.com) (accessed on 1 September 2025). A total of 13 mL of the proliferation medium was transferred into a 75 cm^2^ flask; after that, the thawed cells were transferred into the flask and mixed gently. The flask was labeled and transferred in 5% CO_2_ incubators at 37 °C and 95% humidity.

### 2.2. Differentiation of Osteoblasts Medium

Upon reaching 80–90% confluence, the cells were incubated in an osteoblast differentiation medium that consists of DMEM containing 10% FBS, 1% penicillin-streptomycin, 50 mg/mL L-ascorbic acid, 10 mM b-glycerophosphate (Sigma-Aldrich, www.sigmaaldrich.com, St. Louis, MO, USA, (accessed on 1 September 2025), 10 nM calcitriol (1a,25-dihydroxyvitamin D3; Sigma-Aldrich), and 10 nM dexamethasone (Sigma-Aldrich). Afterward, Bio was added to the medium at a concentration of 3 μM. BIO was initially recognized as a potent suppressor of osteoblastic differentiation in human MSCs, based on a functional screening of a small-molecule inhibitor library, using ALP activity as a read-out, in which the screening was run at a concentration of 3 μM [27,28]. Therefore, the cells were exposed to the small-molecule inhibitor BIO during the differentiation period. As for the control cells, they were cultured in an osteoblast differentiation medium with dimethyl sulfoxide (DMSO) as the control treatment.

### 2.3. Analysis

#### 2.3.1. Assay of Cell Viability

Cell viability assay was performed on day 1, 2, and 3 according to the manufacturer’s (Thermo Fisher Scientific) instructions. In 96-well plates, cells were cultivated and 300 μL of the medium was added to each well with three concentrations, 0.3, 3 and 30 μM, of BIO. For control wells, the medium was treated with DMSO. Then, on days 1, 2, and 3 each well was treated with 30 μL of alamarBlue substrate (10%) and the plates were incubated at 37 °C for 1 h. Readings were taken using fluorescent mode (Ex 530 nm/Em 590 nm) utilizing a BioTek Synergy II microplate reader (BioTek Inc., Winooski, VT, USA).

Moreover, cells were cultured in 24-well plates in 200 μL of the medium and were allowed to proliferate for 10 days. Subsequently, 20 μL/well of 10% alamarBlue substrate was added, followed by 1 h incubation in the dark at 37 °C. Readings were taken using fluorescent mode (Ex 530 nm/Em 590 nm) utilizing a BioTek Synergy II microplate reader (BioTek Inc., Winooski, VT, USA).

#### 2.3.2. Apoptosis Assay

Fluorescence-based analysis was used to measure for apoptotic cells using AO/EB staining to evaluate for dead cells in the 3 μM BIO treated cells compared to DMSO-control-treated ones. The reagent consisted of: acridine orange (100 μL) + Ethedium bromide (170 μL) + 5 mL PBS. On day 3, the medium was removed and the wells were washed with 200 μL of PBS once. The mixture of mentioned reagents was added and mixed for 2 min. Images were taken immediately using a Nikon Eclipse Ti fluorescence microscope (Nikon, Tokyo, Japan).

#### 2.3.3. Quantification of Activity of Alkaline Phosphatase

Alkaline phosphatase (ALP) activity was measured utilizing the BioVision ALP activity colorimetric assay kit (BioVision Inc., Milpitas, CA, USA). First, the cells were cultured in 24-well plates. On day 10, the cells were rinsed once with phosphate-buffered saline (PBS) and fixed with 3.7% formaldehyde in 90% ethanol for 30 s at room temperature. Then, the fixative is going to be removed, and 50 μL of p-nitrophenyl phosphate solution was added to each well, followed by 30–60 min of incubation. Subsequently, optical densities were measured at 405 nm using a fluorescence spectrophotometer plate reader (SpectraMax/M5) (Molecular Devices Co, Sunnyvale, CA, USA), and ALP enzymatic activity in the cell cultures is going to be normalized with respect to the total cell number.

#### 2.3.4. Staining of Alkaline Phosphatase

Staining of differentiated osteoblasts with alkaline phosphatase was performed on day 10 of call culture. In the osteoblast differentiation medium, cells were cultivated in a 24-well plate. The plates were washed in PBS and fixed in 10 mM acetone/citrate buffer at pH 4.2 for 5 min at room temperature. The fixative was then removed, and the Naphthol/Fast Red stain [0.2 mg/mL Naphthol AS-TR phosphate substrate (Sigma)] [0.417 mg/mL of Fast Red (Sigma)] was added for one hour at room temperature. After that, the cells were washed with water and examined under the microscope. Then, the percentage of positive staining area was calculated using ImageJ software (Version 1.53) (U.S. National Institutes of Health, Bethesda, MD, USA). Data are representative of 3 replicas for each BIO concentration.

#### 2.3.5. Alizarin Red S Staining for the Formation of Mineralized Matrix

Alizarin Red staining was conducted on day 14 of osteoblast differentiation. After washing the cells twice with PBL, they were fixed for 15 min at room temperature with 4% paraformaldehyde. Cells were washed with distilled water followed by staining with 2% Alizarin Red S Staining Kit (ScienceCell, Research Laboratories, San Diego, CA, USA, Cat. No. 0223) for 20–30 min at room temperature. Finally, cells can be imaged under the microscope after washing off the dye with water. Then, the percentage of positive staining area was calculated using ImageJ software (Version 1.53) (U.S. National Institutes of Health, Bethesda, MD, USA). Data are representative of 3 replicas for each BIO concentration.

#### 2.3.6. Extraction of RNA and Synthesis of cDNA

Extraction of total RNA from cell pellets is performed utilizing the total RNA Purification Kit on day 10 of osteoblast differentiation following the treatment of hMSC-TERT4 with the 3 µM of BIO. Concentrations of total RNA isolated were measured with NanoDrop 2000 (Thermo Fisher Scientific Inc., Waltham, MA, USA). Then, cDNA was synthesized from 500 ng of total RNA using a High-Capacity cDNA Transcription Kit according to the manufacturer’s instructions. To make cDNA from extracted RNA, each sample consisted of 10 μL of diluted RNA (20 μL RNA + 180 μL nuclease-free H_2_O) add to 10 μL of the reagents mix. The reagent mix consisted of the following (for each sample): 1 μL reverse transcriptase, 2 μL of RT buffer, 0.8 μL of deoxynucleoside triphosphates (dNTP), 2 μL RT primer, and 4.2 μL nuclease-free H_2_O. Samples were then placed in the block of ProFlex PCR System (Thermo Fisher Scientific Inc., Waltham, MA, USA).

Real-time polymerase chain reaction (qPCR) was performed on genes of interest using the synthesized cDNA and utilizing Biosystems ViiA 7 (Thermo Fisher Scientific Inc., Waltham, MA, USA). All primers used in this study are listed in Table 1. For each sample, 5 μL of cDNA was used along with 15 μL of master mix. Master mix consists of the following (per sample): 10 μL Syber Green, 3 μL nuclease-free H_2_O, 1 μL forward primer, and 1 μL reverse primer of the gene of interest. Samples are then loaded in a 96-well plate and placed in the Biosystems ViiA 7 machine. Results were analyzed using Excel, and graphs were generated using GraphPad Prism 6.01.

#### 2.3.7. Gene Expression Profiling by Microarray

On day 10, Quick Amp Labeling Kit (low input) and SurePrint G3 Human GE 8 × 60 k microarray chip were used to label and hybridize 200 ng of total RNA for BIO and DMSO-treated hMSC-TERT4 samples. GeneSpring 13.0 software was used to analyze normalized data. Afterward, pathway analysis was performed using String database 11.5, at a minimum required interaction score of medium confidence (0.400), to demonstrate previously known and predicted interactions between proteins of interest (protein–protein interactions).

#### 2.3.8. Statistical Analysis

Microsoft Excel 2010 and GraphPad Prism 6.01 program (GraphPad, San Diego, CA, USA) were used for statistical analysis and graphing, respectively. Results were indicated as the mean ± SEM of at least two independent experiments. An unpaired *t*-test was used to determine statistical significance, and *p*-value was calculated.

## 3. Results

**Figure 3:**
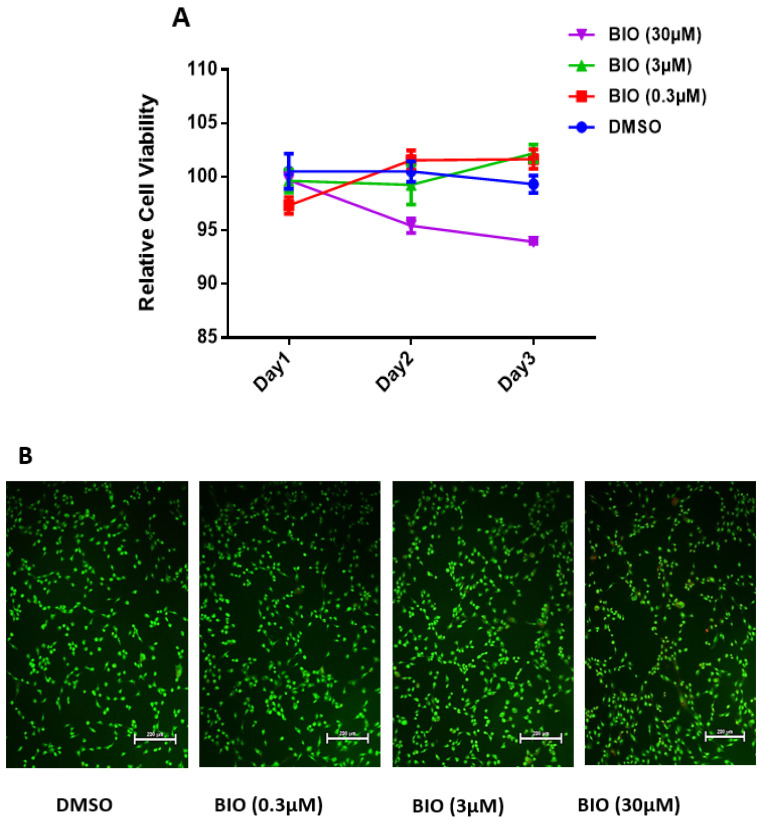
Effects of BIO treatment on the viability of hMSC-TERT4. (**A**) The graph illustrates dose-response proliferation curves of hMSC-TERT4 in response to 0.3 μM, 3 μM, and 30 μM concentrations of BIO compared to DMSO-treated cells utilizing cell viability assay (alamarBlue) over the course of 3 days. (**B**) On day 3, the apoptosis assay carried out for BIO-treated hMSC-TERT4 (0.3 μM, 3 μM, and 30 μM concentrations) compared to the DMSO control. Images were taken at 10× Magnification using a Nikon Eclipse Ti fluorescent microscope; apoptotic cells indicated by white circles.

To study the toxicity of BIO on hMSC-TERT4, alamarBlue assay was used in a dose–response fashion over three days. AlamarBlue assay, a fluorometric method to measure cellular metabolic activity to ensure cell viability. First, we evaluated the effect of the in vitro treatment of BIO at a logarithmic scale at concentrations of 0.3, 3, and 30 µM for 1, 2, and 3 days on hMSC-TERT4 proliferation (Figure 3A). Both 0.3 and 3 µM concentrations confirmed no significant effects of BIO on hMSC-TERT4 proliferation, while the concentration of 30 µM demonstrated a significant adverse effect on cell proliferation (Figure 3A). For a better apoptosis elucidation, flow cytometry could be a valuable addition in future work. Furthermore, an apoptosis assay was carried out on day 3, following 0.3, 3, and 30 µM treatment of BIO to rule out activation-induced cell death (Figure 3B). No significant differences in the number of apoptotic cells were detected at 0.3 and 3 μM concentrations compared to the DMSO-treated control, whereas treatment of 30 μM concentration detected apoptotic and necrotic cells compared to the DMSO-treated control cells (Figure 3B).

### 3.1. BIO Downregulates Osteogenic Differentiation of hMSC-TERT4

**Figure 4:**
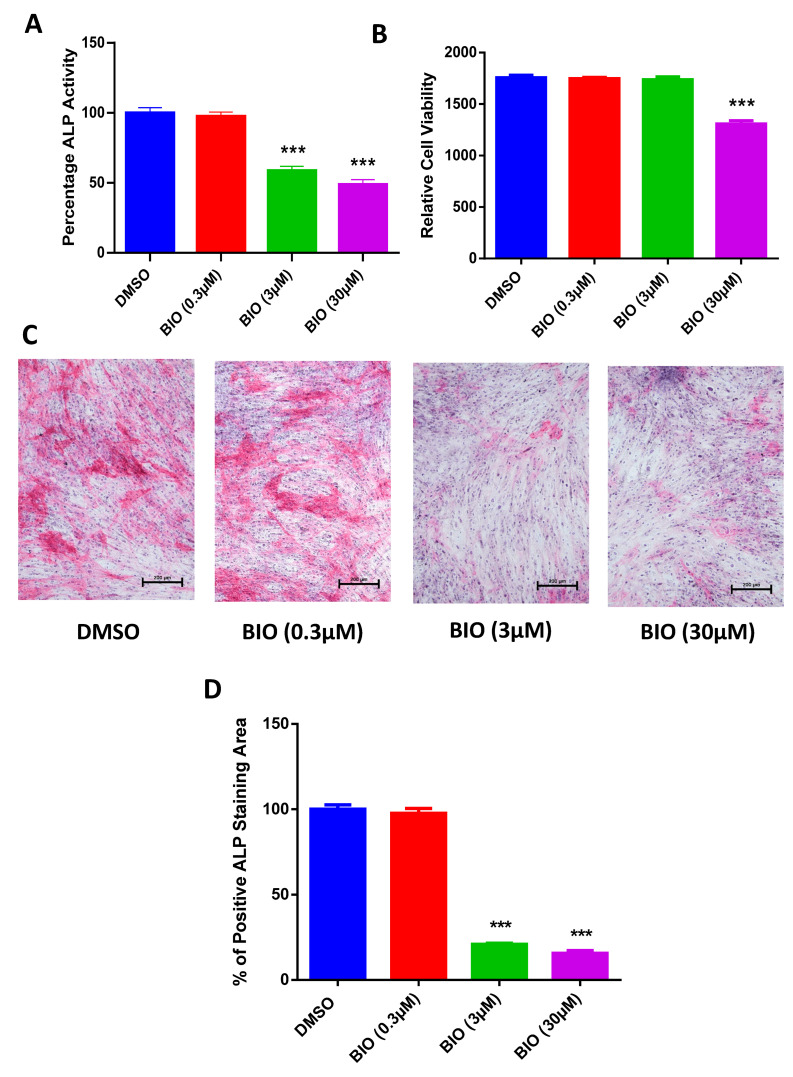
**Influence of BIO on osteoblastic differentiation of hMSC-TERT4** (**A**) The graph demonstrates quantitative ALP activity at day 10 post osteoblast differentiation induction and exposure to BIO and DMSO control (*n* = 20), *p*-value: *** *p* < 0.0005. (**B**) Day 10 post osteoblast differentiation induction and exposure to BIO and DMSO control cell viability is carried out with no significant difference between 3µM BIO and DMSO control (*n* = 20), *p*-value: *** *p* < 0.0005. (**C**) Images show cytochemical ALP staining of hMSC-TERT4 on day 10 post osteoblast differentiation induction and exposure to BIO at 0.3 µM, 3 µM, and 30 µM concentrations compared to DMSO control. (**D**) Percentage of positive Cytochemical ALP staining area of hMSC-TERT4 on day 10 post osteoblast differentiation induction and exposure to BIO at 0.3 µM, 3 µM, and 30 µM concentrations compared to DMSO control.

The assay was performed at day 10 post osteoblast differentiation induction where the 0.3 and 3 μM BIO treatments showed no detectable influence on hMSC-TERT4 viability, while 30 μM BIO treatment showed signinficant decrease in cell viability (Figure 4B). Furthermore, only BIO-treated hMSC-TERT4 at 3 μM and 30 μM show a significant reduction in ALP activity (Figure 4A). The effect was supported by the reduction in ALP cytochemical staining compared to DMSO control illustrated by the stained cytoplasm of the cells (Figure 4C), and the percentage of positive staining area was further measured and showed statistical decrease in BIO-treated hMSC-TERT4 at 3 μM and 30 μM concentrations (Figure 4D).

### 3.2. Effects of BIO Treatment on hMSC-TERT4 In Vitro

**Figure 5:**
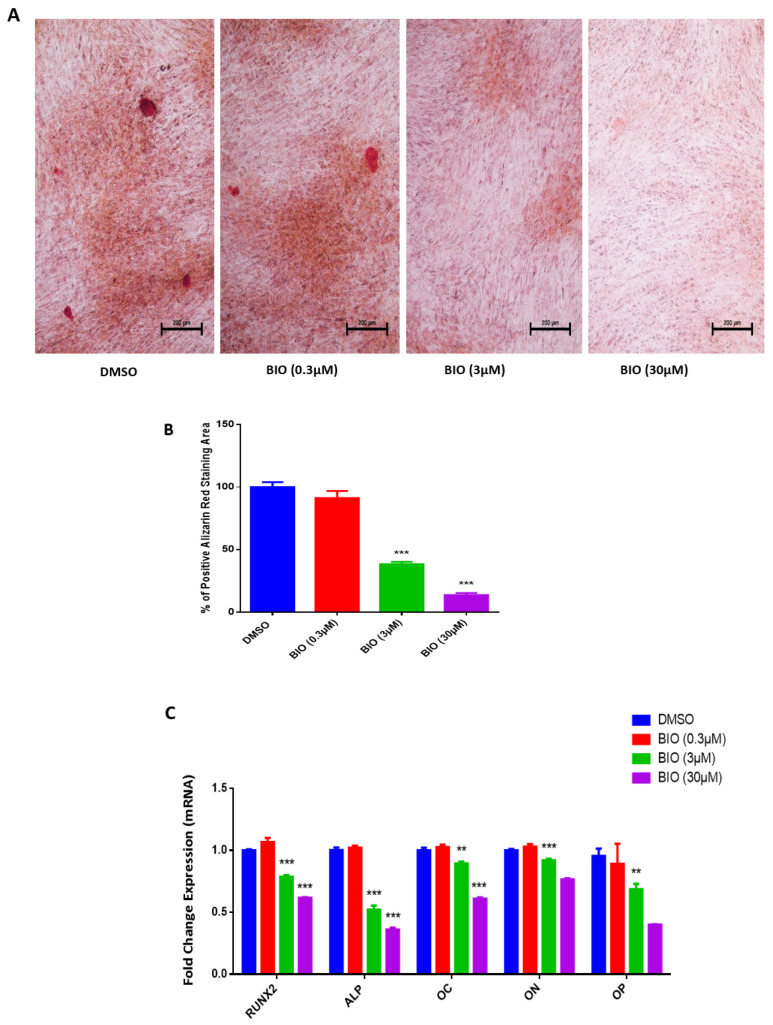
**Effects of BIO treatment on hMSC-TERT4 in vitro.** (**A**) Depicts mineralized matrix changes using Alizarin Red stain obtained on day 14 post osteoblast differentiation induction and exposure to BIO at 0.3 µM, 3 µM, and 30 µM concentrations compared to DMSO control at 10× Magnification. (**B**) Percentage of positive Alizarin Red staining area on day 14 post osteoblast differentiation induction and exposure to BIO at 0.3 µM, 3 µM, and 30 µM concentrations compared to DMSO control. (**C**) Quantitative RT-PCR analysis results on day 10 post osteoblast differentiation induction. RT-PCR two independent runs (*n* = 6) from two independent experiments; *p*-value: ** *p* < 0.005; *** *p* < 0.0005. Abbreviations: RUNX2—Runt-related transcription factor 2; ALP—alkaline phosphatase; OC—Osteocalcin; ON—Osteonectin; OP—Osteopontin; DMSO—dimethyl sulfoxide.

We performed Alzarin red staining as an indicator of mineralization and matrix formation to further confirm the previous result. Calcium deposits (mineralized matrix) formation is demonstrated in Alizarin red staining post 0.3 μM BIO treatment of hMSC-TERT4 on day 14, while no calcium deposits are shown in 3 μM or 30 μM BIO treatments compared to DMSO control indicating reduced osteoblast differentiation (Figure 5A). Percentage of positive Alizarin Red staining area was further measured and showed statistical significant reduction post 3μM and 30 μM BIO treatments of hMSC-TERT4 on day 14 (Figure 5B).

Gene expression of osteoblast differentiation markers was performed 10 days post osteoblast differentiation induction and exposure to BIO at 0.3 µM, 3 µM, and 30 µM concentrations. While 0.3 µM treatment demonstrated no significant difference in all osteoblast differentiation markers compared to DMSO control (Figure 5C), 3 µM showed significant reduction in the genetic expression of osteoblast differentiation markers; RUNX2, ALP, Osteocalcin (OC), Osteonectin (ON), and Osteopontin (OP), compared to DMSO control (Figure 5C). Differentiated hMSC-TERT4 treated with 30 µM BIO exhibited significant reduction in RUNX2, ALP, and OC only, with no significant difference in ON and OP (Figure 5C). Based on all the above data, only BIO treatment at 3 µM concentration was used for further investigations of this study.

### 3.3. BIO Affects Multiple Signaling Pathways During Osteoblast Differentiation of hMSC-TERT4

**Figure 6:**
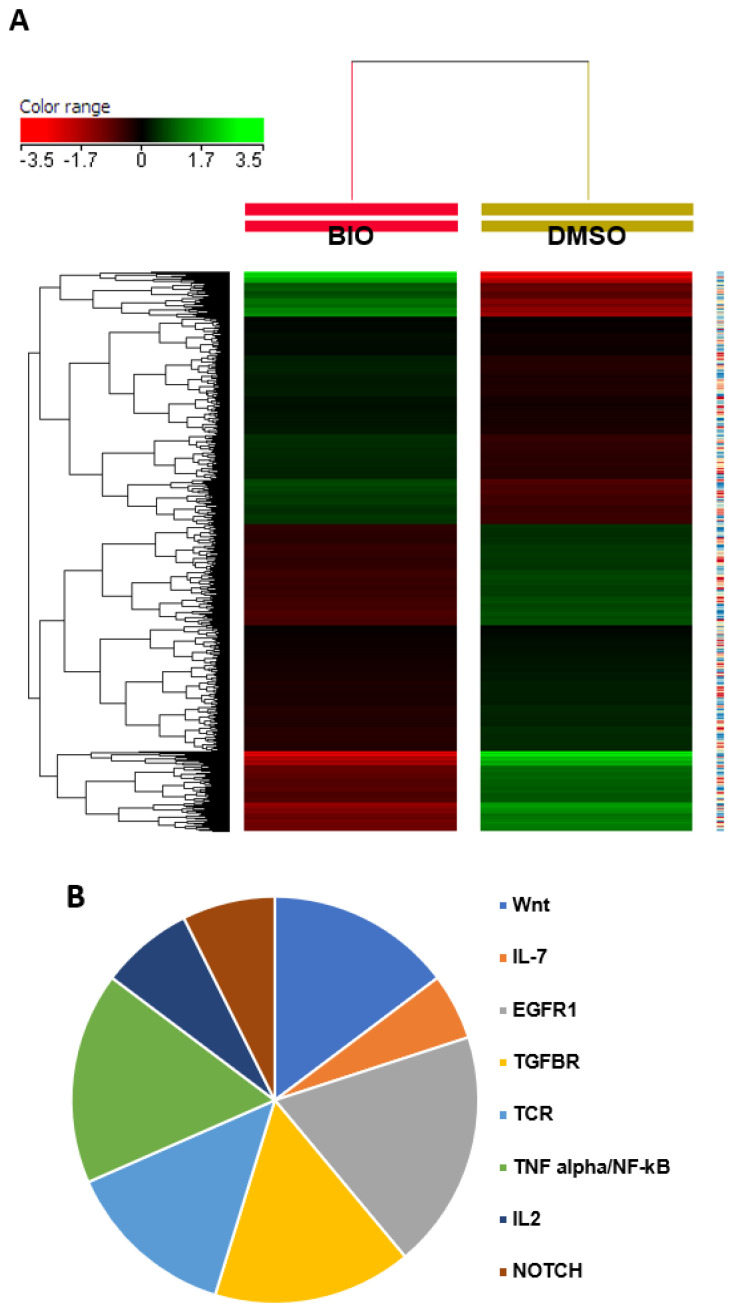

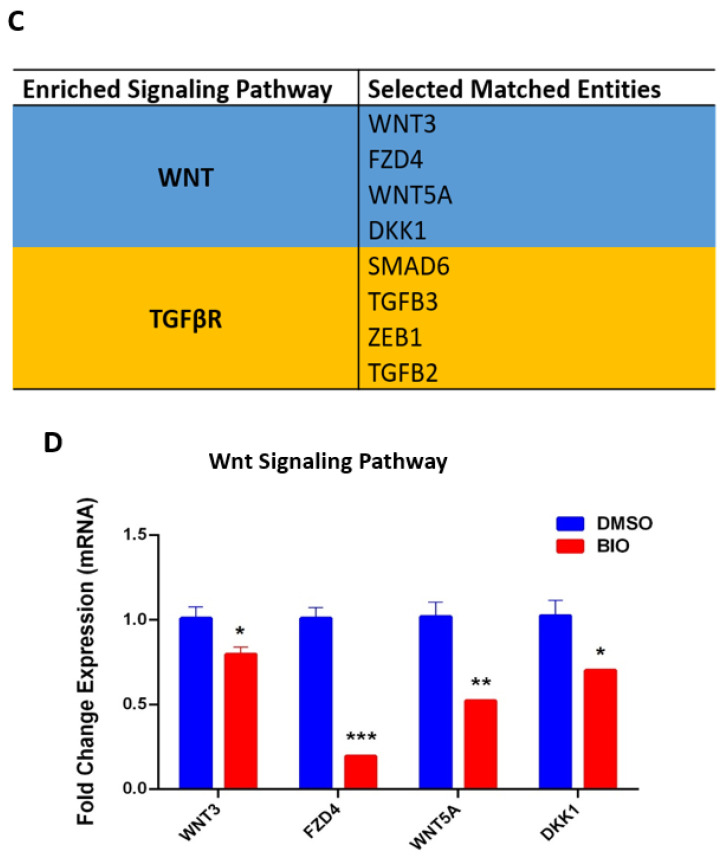

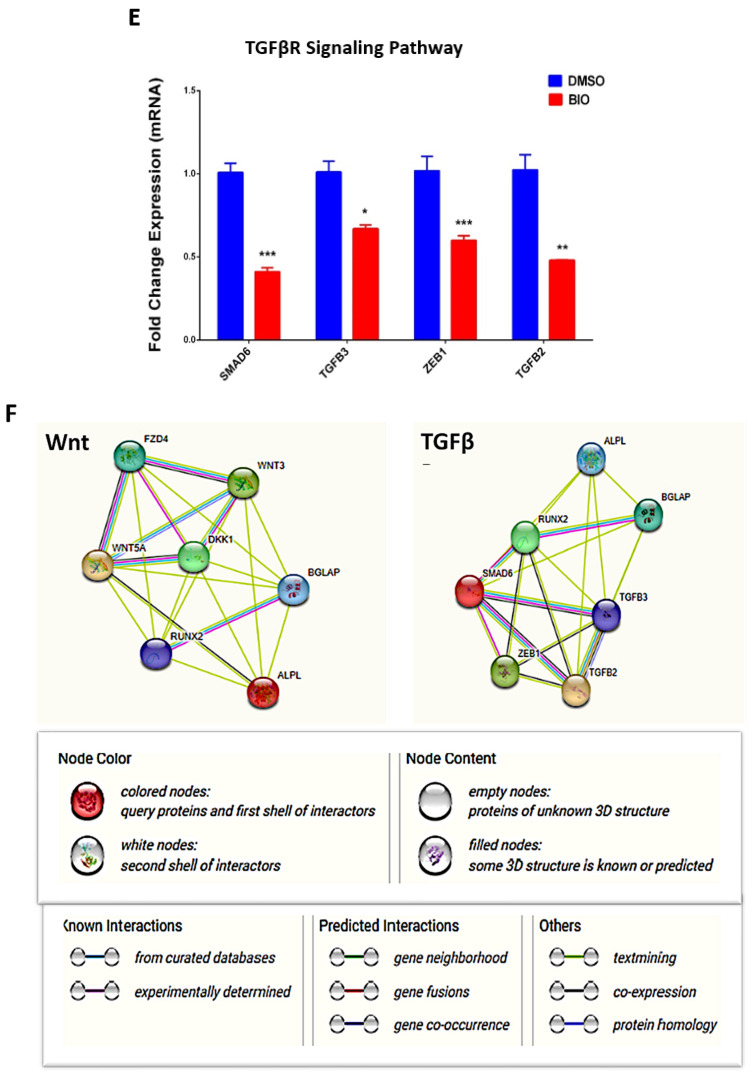
**BIO affects multiple pathways during hMSC-TERT4 differentiation into osteoblasts.** (**A**) Microarray analysis shows unsupervised hierarchical clustering of BIO-treated compared to DMSO-treated control differentially expressed mRNA levels. (**B**) The pie chart shows the top 8 enriched signaling pathways based on identified differentially expressed genes in the BIO-treated hMSC-TERT4 compared to the DMSO-treated hMSC-TERT4 control. The size of each segment of the pie charts represents fold enrichment. (**C**) The table demonstrates selected enriched signaling pathways and their matched entities for qRT-PCR. (**D**) Real-time PCR results for selected genes of Wnt and (**E**) TGFβR enriched signaling pathways (*n* = 6) from two independent experiments; *p*-value: * *p* < 0.05; ** *p* < 0.005; *** *p* < 0.0005. (**F**) Bioinformatics of selected enriched pathway genes for Wnt and TGFβR with osteoblast gene markers.

A Global gene expression profiling of BIO-treated hMSC-TERT4 and pathway analysis was done to further understand the molecular mechanisms by which BIO reduces differentiation compared to DMSO-treated hMSC-TERT4 control cells. Microarray analysis shows a clear separation on the unsupervised hierarchical clustering performed of BIO-treated compared to DMSO-treated control based on differentially expressed levels of mRNA (Figure 6A). Pathway analysis was carried out for the downregulated genes, represented by a pie chart for the top 8 enriched signaling pathways (Figure 6B).

Two enriched signaling pathways are selected (Wnt and TGFβR), with four matched entities each, and validated using qRT-PCR. The results of the qRT-PCR for selected Wnt (WNT3, FZD4, WNT5A, and DKK1) and TGFβR (SMAD6, TGFβ3, ZEB1, and TGFβ2) signaling pathway genes collectively were significantly downregulated, confirming the microarray results (Figure 6D,E). The functional enrichment analysis performed demonstrates protein–protein interaction between osteoblast proteins (ALP, RUNX2, and Osteocalcin) and the selected Wnt and TGFβR proteins (WNT3, FZD4, WNT5A, DKK1, SMAD6, TGFβ3, ZEB1, and TGFβ2), supported by experimentally determined data and curated using String database (Figure 6F). A limitation of this study is the absence of protein-level validation of those key signaling pathway genes. While our findings demonstrate consistent functional enhancement analysis and coordinated regulation of osteogenic genes, protein-based analyses such as Western blotting for β-catenin and SMAD family members would further strengthen the mechanistic interpretation. This will be addressed in future studies.

## 4. Discussion

In the present study, treatment of hMSC-TERT4 with BIO resulted in a marked suppression of osteogenic differentiation, demonstrated by reduced ALP activity, diminished matrix mineralization, and downregulation of osteoblast-associated transcripts including RUNX2, ALP, OCN, ON, and OP. Gene expression profiling and pathway analysis further revealed downregulation of Wnt/β-catenin and TGF-β signaling, two pathways essential for osteoblast lineage commitment and maturation [4,29]. These findings contrast with the osteogenic enhancement typically observed following selective GSK3 inhibition, which stabilizes β-catenin and supports RUNX2-mediated transcriptional activity [17,30]. The inhibitory phenotype observed may reflect additional kinase targets of BIO, including reported JAK inhibition.

While GSK3 inhibition is generally expected to enhance osteoblastogenesis through β-catenin activation, BIO is also a potent pan-JAK inhibitor [8,31]. JAK/STAT signaling is indispensable for cytokine-driven osteoblast maturation, particularly through IL-6/LIF-mediated STAT3 phosphorylation, which promotes RUNX2 and Osterix activity [32,33]. Disruption of STAT3 signaling is known to impair osteoblast differentiation and reduce mineralization [5,6]. Moreover, GSK3 itself modulates STAT activity, with GSK3 inhibition increasing STAT3 serine phosphorylation while reducing its transcriptional capacity [34]. Taken together, these interactions highlight a highly integrated network wherein BIO’s dual inhibition may shift pathway dominance toward a net anti-osteogenic effect.

Our microarray data of pathway analysis also showed significant downregulation of TGF-β–associated transcripts (TGFβ2, TGFβ3, SMAD6, ZEB1). This is noteworthy because TGF-β supports early osteoblast commitment through Smad2/3-guided induction of RUNX2 [35] and interacts closely with JAK/STAT signaling during mesenchymal lineage specification [36]. While TGF-β exhibits stage-dependent inhibitory effects in late mineralization, suppression of early TGF-β/Smad activity is known to impair osteoprogenitor progression [37]. Thus, the coordinated inhibition of both JAK/STAT and TGF-β pathways by BIO likely contributes significantly to the reduction in osteogenic potential observed in our study. Moreover, the bioinformatics of the selected enriched pathway genes for Wnt and TGFβR with osteoblast gene markers offers a useful visualization of known associations between osteogenic markers and Wnt and TGFβR pathway genes. However, it is a database-derived and does not assess functional pathway activity in an experimental setting, which remains a limitation.

The inhibitory effect identified here aligns with previous reports showing that BIO exhibits dose-dependent and context-dependent actions [38,39,40]. While low concentrations (<1 μM) may enhance osteogenesis by partially activating Wnt signaling [40], higher concentrations of BIO suppress proliferation, migration, and differentiation, largely due to JAK inhibition and cell cycle disruption [38,39]. JAK inhibitors such as Tofacitinib and Ruxolitinib have similarly been shown to reduce bone formation and impair osteoblast function in vivo and in vitro [27,41]. Thus, our concentration of 3 μM BIO falls within a range known to shift the biological response from pro-osteogenic to anti-osteogenic, consistent with a dominant JAK-suppressive effect.

Although BIO is widely used as a GSK3 inhibitor, it also targets additional kinases such as CDKs, CLK, and DYRK family members [42,43]. Such off-target interactions may influence cell cycle progression or stress-response pathways, potentially contributing to reduced osteogenic output independent of canonical JAK/STAT or Wnt signaling [16,44]. Importantly, reduced osteogenesis in our study cannot be attributed to cytotoxicity, as cell viability remained sufficient to support differentiation. Nevertheless, the complexity of BIO’s kinase-binding profile highlights the limitations of attributing phenotypes to GSK3 inhibition alone.

The suppression of osteogenesis by BIO has important implications for regenerative medicine and bone-tissue engineering. Given that several JAK inhibitors, such as Tofacitinib, are clinically used immunomodulators known to impair bone formation [45], our findings reinforce the need for caution when applying multi-targeted kinase inhibitors in MSC-based therapies. Therapeutic strategies aiming to enhance osteogenesis must consider the opposing effects of JAK suppression, even when compounds simultaneously activate pro-osteogenic pathways such as Wnt/β-catenin. Understanding this balance may guide the development of more selective modulators for skeletal repair. Although the hMSC-TERT4 cell line retains the key features and functional characteristics of primary hMSCs, its immortalized nature may limit direct physiological generalization. Telomerase-mediated immortalization can alter cell cycle regulation, stress responses, or signaling pathways, which may influence cellular behavior under experimental conditions. Therefore, these findings may not fully capture primary hMSCs or in vivo settings [46].

## Figures and Tables

**Table 1:** List of Syber Green primers.

Gene Name	Forward Primer	Reverse Primer
ACTB	AGCCATGTACGTTGCTA	AGTCCGCCTAGAAGCA
ALP	GACGGACCCTCGCCAGTGCT	AATCGACGTGGGTGGGAGGGG
RUNX2	GTAGATGGACCTCGGGAACC	GAGGCGGTCAGAGAACAAAC
OC	CTCACACACCTCCCTCCTG	GGCAGCGAGGTAGTGAAGAG
ON	GAGGAAACCGAAGAGGAGG	GGGGTGTTGTTCTCATCCAG
OP	CAGTTCAGAAGAGGAGG	TCAGCCTCAGAGTCTTCATC
WNT3	ATGCGCGCGAGAACAGG	TGGTCCAGGATAGTCGTGC
FZD4	GCTGACAACTTTCACACCGC	AACAGAACAAAGGAAGAACTGC
WNT5A	TCGCTCCGCTCGGATTC	AATATTCCAATGGACTTCTTCATGG
DKK1	TGACAACTACCAGCCGTACC	GGGACTAGCGCAGTACTCATC
SMAD6	GGGCCCGAATCTCCGC	AATCGGACAGATCCAGTGGC
TGFB3	ATTCCGAGCAGAATTCCGGG	CTGGCCGAAGGATCTGGAAG
ZEB1	GTCACTTCCCATCCCGGTTC	AAAGGCGACGGGCTGAC
TGFB2	GCGACGAAGAGTACTACGCC	GCGGGATGGCATTTTCGGAG

## Data Availability

The original contributions presented in this study are included in the article. Further inquiries can be directed to the corresponding author.

## Abbreviations

MSCs	Mesenchymal stem cells
hMSCs	Human mesenchymal stem cells
Human mesenchymal stem cells	Glycogen-synthase-kinase-3
JAK	Janus kinase
STAT	signal transducer and activator of transcription
STAT3	signal transducer and activator of transcription 3
BIO	6-bromoindirubin-3′-oxime
IL-6	interleukin 6
ALP	Alkaline phosphatase
DMEM	Dulbecco’s modified Eagle’s medium
DMSO	Dimethyl sulfoxide
hTERT	Human telomerase reverse transcriptase
PBS	Phosphate-buffered saline
qRT-PCR	Quantitative reverse transcriptase polymerase chain reaction
BMP	Bone morphogenetic proteins; Runx2
Runx2	Runt-related transcription factor 2
OC	Osteocalcin
ON	Osteonectin
OP	Osteopontin
Wnt	Wingless/int1
TGF-β	Transforming growth factor β
TGF-βR	TGF-β receptor
SMAD	Mothers against decapentaplegic homolog
SMAD6	Mothers against decapentaplegic homolog 6
Wnt3	Wingless/int1 3
Wnt5A	Wingless/int1 5A
FZD4	Frizzled class receptor 4
DKK1	Dickkopf-related protein 1
ZEB1	Zinc finger E-box binding homeobox 1
TGF-β2	Transforming growth factor β2
TGF-β3	Transforming growth factor β3
